# Bone, muscle, and physical function measures in older adults according to levels of social disadvantage: a cross-sectional study

**DOI:** 10.1007/s11657-026-01661-5

**Published:** 2026-02-01

**Authors:** Jason Talevski, Sharon Brennan-Olsen, Stefanie Bird, Sara Vogrin, Alison Beauchamp, Mizhgan Fatima, Cassandra Smith, Gustavo Duque

**Affiliations:** 1 School of Psychology and Public Health, La Trobe University, Melbourne, Victoria Australia; 2 Australian Institute for Musculoskeletal Science (AIMSS), The University of Melbourne and Western Health, St Albans, , Victoria Australia; 3 The University of Melbourne, Melbourne, Victoria Australia; 4 Institute for Physical Activity and Nutrition Research (IPAN), School of Exercise and Nutrition Sciences, Deakin University, Geelong, Victoria Australia; 5 The GrantEd Group, Bentleigh, Victoria Australia; 6 School of Rural Health, Monash University, Warragul, Victoria Australia; 7 Victorian Heart Institute, Monash Health, Clayton, Victoria Australia; 8 Nutrition and Health Innovation Research Institute, School of Medical and Health Sciences, Edith Cowan University, Joondalup, Western Australia Australia; 9 Medical School, The University of Western Australia, Perth, Western Australia Australia; 10 Bone, Muscle & Geroscience Group, Research Institute of the, McGill University Health Centre, McGill University, Montreal, Canada

**Keywords:** Bone, Muscle, Osteoporosis, Physical function, Sarcopenia, Social disadvantage

## Abstract

**Summary:**

This cross-sectional study of 300 older adults (aged ≥ 50 years) found that less education, lower income, and health care card ownership are associated with reduced bone, muscle, and physical function measures. This underscores the need for targeted preventive strategies for osteoporosis and sarcopenia that address socioeconomic-related disparities.

**Purpose:**

The prevalence of chronic diseases follows a social gradient, although this is unclear in musculoskeletal conditions. This study aims to examine the association between social disadvantage and diagnostic measures of osteoporosis and sarcopenia in community-dwelling older adults.

**Methods:**

A single-centre, cross-sectional study was conducted in adults (≥ 50 years) residing in the metropolitan region of Melbourne, Australia. Data on socio-demographic variables were collected via self-reported questionnaires. Social disadvantage variables included education, income, employment status, health care card ownership, and area-level socioeconomic status. Outcomes of interest were bone mineral density (BMD), appendicular lean body mass (ALM/h^2^), hand grip strength, lower limb strength, and physical performance (gait speed; short physical performance battery; leg power). Multivariable linear regression was used to analyse associations between social disadvantage and outcome measures.

**Results:**

A total of 300 participants were recruited (mean age: 66.8 years; 61.7% female). Post-secondary education was associated with higher BMD (β = 0.29; 95% CI: 0.01–0.58), ALM/h^2^ (β = 0.29; 95% CI: 0.08–0.51), handgrip strength (β = 2.25; 95% CI: 0.57–4.52), gait speed (β = 0.08; 95% CI: 0.01–0.15), and leg power (β = 41.7; 95% CI: 11.3–71.9). Positive associations in these outcomes were also correlated with higher income and not having a health care card. Employment status and area-level socioeconomic status showed limited associations with most outcomes.

**Conclusion:**

This study demonstrates that social disadvantage is associated with poorer bone, muscle, and physical function in older adults. Targeted interventions that address socioeconomic-related disparities may support effective prevention of osteoporosis and sarcopenia later in life.

## Introduction

Worldwide, musculoskeletal disorders are the second greatest cause of disability among adults [1]. Osteoporosis and osteopenia—characterised by low bone mineral density (BMD)—are one of the most prevalent types of musculoskeletal conditions among people aged ≥ 50 years [2]. A recent meta-analysis of 70 studies estimated the prevalence of osteoporosis to be approximately 18.3% in older adults (23.1% in women and 11.7% in men) [3]. Fragility fractures are the most severe clinical outcome of osteoporosis and can lead to long-term functional impairment [4], reduced quality of life [5, 6], and premature mortality [7, 8]. Historical estimates show that osteoporosis results in a fragility fracture occurring every 3 s globally, and the global incidence of hip fractures increased 2.6-fold from 1990 to 2019 [9]. Sarcopenia, also a common musculoskeletal condition, encompasses the progressive age-related loss of muscle mass and decline in strength and physical function [10]. Sarcopenia affects between 10 and 27% of individuals aged ≥ 60 years [11], and like osteoporosis, it is associated with a high burden of morbidity and mortality [12].

The prevalence of chronic diseases follows a social gradient, whereby higher incidence is observed in populations with lower socioeconomic status (SES) [13, 14]. Lower SES populations also have higher rates of healthcare utilisation compared to their higher SES counterparts [15, 16] and also face challenges in accessing alternative healthcare approaches such as virtual care [17]. There is growing recognition of the influence of SES on musculoskeletal health. Social disadvantage increases exposure to cumulative stressors and inflammatory responses, which, alongside lifestyle factors and biological mechanisms, contribute to a heightened risk of fractures [18].

Data from population-based studies have consistently shown that lower SES—measured by income, education, or area-level disadvantage—is associated with reduced BMD and increased risk of fracture [19–21]. However, the impact of SES on sarcopenia prevalence is not well understood. In an Australian study, individuals with higher educational attainment were associated with stronger grip and leg strength, and greater muscle mass [22]. A recent cross-sectional study in a large sample of community-dwelling older adults in the UK reported that disadvantaged socioeconomic position, defined by educational attainment and subjective social status, was associated with an increased likelihood of probable sarcopenia when controlling for other known risk factors [23].

While these studies provide context and suggest a need to address socioeconomic disadvantage for sarcopenia prevention strategies, neither was specifically designed to examine the relationship between SES and sarcopenia as a primary aim, relying on secondary analyses of existing longitudinal datasets. Additionally, neither study incorporated a comprehensive range of individual-level measures of SES, highlighting the need for dedicated studies that explicitly investigate the association between SES and sarcopenia. This study aims to examine associations between parameters of social disadvantage and diagnostic measures of osteoporosis and sarcopenia.

## Methods

### Study design and setting

A single-centre, cross-sectional study was conducted at Sunshine Hospital (Western Health), located in the Western metropolitan region of Victoria, Australia. This health service was selected to represent a diverse range of socioeconomic and geographical characteristics of people living within Victoria.

### Participants

Individuals living in the western and surrounding suburbs of Victoria were recruited between January 2018 and March 2020. The primary recruitment strategy was through poster advertisements at Sunshine Hospital, community groups and services, general practice clinics in the western suburbs, local community centres, and local support groups. Individuals who enquired about participating in the study were screened for eligibility (Box 1) by a research assistant via telephone. After the screening process, eligible individuals were provided with the participant explanatory statement, and written consent was obtained. An appointment was then made for participants to attend a data collection session within the following 4 weeks.

### Data collection and study outcomes

All physical outcome assessments were undertaken at the Falls and Fracture Clinic, located in Sunshine Hospital, Melbourne. Each session lasted approximately 2 h. Data on socio-demographic variables, health behaviours and general health outcomes were collected by self-reported surveys. Participants were provided with a self-report questionnaire booklet for completion at (or within 7 days of) their assessment.

#### Demographic variables and socioeconomic position

Self-reported data were collected on demographic variables (age, sex, marital status, lives alone, Aboriginal and/or Torres Strait Islander origin), socioeconomic variables (highest level of education, income, employment status, postcode, health care card), and cultural and linguistic variables (country of birth, primary language spoken at home). Comorbidities were self-reported by participants using a structured questionnaire based on the Charlson Comorbidity Index, although osteoporosis and sarcopenia were assessed using objective measures. BMD was measured using a whole-body dual-energy X-ray absorptiometry (DXA) scan, and osteoporosis was defined according to World Health Organization (WHO) criteria as a BMD T-score ≤ −2.5 [2], using the lowest value from the lumbar spine, femoral neck, or distal radius. Sarcopenia was defined using the European Working Group on Sarcopenia in Older People (EWGSOP2) revised criteria for sarcopenia. Probable sarcopenia was identified by low muscle strength (handgrip strength < 27 kg in men and < 16 kg in women) and/or a five-times sit-to-stand test time > 15 s. Area-level SES was assessed by linking each participant’s residential postcode to the corresponding 2021 Australian Bureau of Statistics (ABS) Census Collection District. The Socio-Economic Indexes for Areas (SEIFA), generated using ABS software, were then used to assign SES values. SEIFA comprises four separate indices developed from Census data, each reflecting different aspects of area-level advantage and disadvantage [24]. For this study, the Index of Relative Socioeconomic Advantage and Disadvantage (IRSAD) was used, which comprises measuring both social advantage and disadvantage. IRSAD incorporates variables such as income, education, employment, and housing to create a multidimensional SES profile. Participants were assigned a score of social disadvantage according to predetermined deciles for SEIFA values (1–10). A low score represents a more disadvantaged area [24]. IRSAD scores were then divided into quintiles (1 = most disadvantaged, 5 = least disadvantaged), in line with previous research.

**Table:** **Box 1 **Study inclusion and exclusion criteria

Inclusion criteria		
1. Aged 50 years or above.		
2. Ability to attend Sunshine Hospital for study assessments.		
3. Able to provide informed consent (e.g., not cognitively impaired).		
Exclusion criteria		
1. Body weight ≥ 150kg (a contraindication for undergoing dual energy x-rayabsorptiometry [DXA] or peripheral quantitative computerised tomography [pQCT] scans).		
2. Non-English speaking.		
3. Previous stroke, hip or knee replacement, spinal surgery, myocardial infarction or majorheart surgery (last 6 months).		
4. Having a health condition that would interfere with ability to complete functional tests (e.g., Parkinson’s Disease, multiple sclerosis).		
5. Living in a residential aged care facility.		

#### Anthropometry

Height was measured to the nearest 0.1 cm using a portable Seca stadiometer, and weight was measured to the nearest 0.1 kg using a single pair of calibrated electronic scales (Seca Delta Model 707, Hamburg, Germany). Body mass index (BMI) was subsequently calculated as kg/m^2^. A 12 ml blood sample was drawn from consenting participants by a qualified technician to collect levels of high-sensitivity C-reactive protein (hs-CRP) and vitamin D.

#### Body composition and bone mineral density (BMD)

Whole body composition was determined by a DXA scan (Hologic Inc, Bedford, MA) by a trained technician. Appendicular lean body mass (ALM; sum of lean mass in the arms and legs) was assessed and corrected for height squared (ALM/h^2^). BMD (g/cm^2^) at the femoral neck, lumbar spine, and wrist was derived using computer algorithms provided by the manufacturer.

#### Muscle strength

Hand grip strength was assessed in both hands using a Jamar hydraulic hand grip dynamometer (Lafayette Instrument Company, USA), which is the gold standard for hand grip strength measurement [25]. The participant was asked to hold the hand grip dynamometer with their elbow at a 90° angle and their opposite arm resting on their lap, then asked to apply as much force as possible for 3 seconds while in a seated position. This test was completed three times with a 30-seconds rest between trials. The highest measure from the three trials was used as the study measure for hand grip strength.

Lower limb strength was measured using the five-times sit-to-stand test. The participant was instructed to attempt to stand up straight from a seated position five times as quickly as possible, without stopping in between and keeping their arms folded across their chest. The time taken to complete the test and/or the number of stands completed were recorded. The test was aborted after 1 min (maximum score of 60 seconds given). 

#### Physical function

Gait speed was assessed using the 4-meter gait speed (m/second). A walking course was marked out on the floor, and the participant was instructed to walk from one end of the course to the other at a comfortable pace. The stopwatch was started as the participant started walking and stopped once the participant completed the course. Three trials (one familiarisation, two testing) were performed, with the fastest of the latter two trials used for analyses. 

The Short Physical Performance Battery (SPPB) is a highly validated measure of physical performance and disability in older adults and is widely used in clinical and research settings [26]. A SPPB summary score of 0 to 12 (higher score indicating better function) is obtained based on performance in three tasks of walking speed, chair rises, and standing balance.

Leg power, which is meaningfully associated with mobility performance, was assessed using the clinically relevant and reliable stair climb power test [27]. Participants were instructed to safely ascend a 10-stair flight of stairs as fast as they could, using the handrail if needed. Stair climb time was recorded to the nearest 0.01 s, and the highest score of two trials was calculated. Stair climb power was calculated with the following formula: *Power (watts)* = *Force × Velocity*. The vertical height of the stairs (1.73 m) and stair climb time (seconds) were used to calculate velocity (distance/time), and the participant’s body mass (kg) and the acceleration due to gravity (9.8m/s^2^) were used to calculate force.

### Statistical analyses

Descriptive statistics and frequencies were used to describe participant characteristics, presented as mean and standard deviation (continuous variables) or frequency and percentage (categorical variables). Normality of continuous variables was evaluated using histograms and the Shapiro–Wilk test. Linear regression modelling was used to analyse associations between social disadvantage and measures of bone, muscle, and physical function, presented as coefficients (mean differences) with 95% confidence intervals (CIs). SES variables were dichotomised where appropriate to perform the analyses (e.g. education was separated into secondary school or less vs. post-secondary school education; IRSAD scores were separated into bottom 5 deciles vs. top 5 deciles). Because of the potential for confounding, multivariable linear regression models were undertaken. Confounding variables were selected based on their significant associations with outcomes in univariate analyses. The variance inflation factor (VIF) was used to examine collinearity among variables in each model, where a VIF result surpassing five was considered indicative of problematic collinearity, which resulted in this variable being removed from the final model [28]. Variables tested for inclusion in final models included age, sex, Australian-born status, Indigenous status, BMI, smoking status (ever or current), alcohol intake, hs-CRP level, and vitamin D level. To minimise overfitting, each confounder was adjusted individually in separate models, although age and sex were included in the final multivariable models regardless of a significant association to account for any latent confounding effects that may exist. A *p*-value of < 0.05 was considered statistically significant. All analyses were performed using STATA statistical software, version 18.5 (Stata Corporation, Inc., College Station, TX, USA).

## Results

### Participants

During the recruitment period, 412 individuals were screened. Of these, a total of 300 participants were found eligible and consented to the study. The study cohort had a mean age of 66.8 years, and there were a higher number of female participants (*n* = 185; 61.7%). The majority of participants were born in Australia (*n* = 209; 69.7%) and were living with someone (*n* = 232; 77.3%). Participants reported a high incidence of comorbidities, with the most common being arthritis (34.2%) or chronic back pain (31.9%). In terms of socioeconomic variables, over half the participants had completed a post-secondary/tertiary qualification (*n* = 156; 52.3%); the majority of participants reported their income level within the two lowest brackets (*n* = 166; 55.7%); most participants were retired or not working at the time of the study (*n* = 205; 68.8%); and there were 128 participants (42.7%) residing in the lowest five SES deciles in Victoria. Table 1 summarises participant demographic and socioeconomic characteristics.

**Table 1:** Demographic characteristics of participants (*n* = 300)

	***N*** (%)
Age, mean ± SD	66.8 ± 8.0
Sex (female)	185 (61.7)
Country of birth	
Australia	209 (69.7)
UK	28 (9.3)
Italy	7 (2.3)
New Zealand	5 (1.7)
Other	51 (17.0)
Lives alone	68 (22.7)
Education (***n*** = 298)	
Completed some or all primary school	11 (3.7)
Completed some or all secondary school	67 (22.5)
Completed some post-secondary school/tertiary qualification	64 (21.5)
Completed all post-secondary/tertiary qualification	156 (52.3)
Employment status (***n*** = 298)	
Full-time	29 (9.7)
Part-time/casual	64 (21.5)
Volunteer	52 (17.4)
Home-based duties/carer	11 (3.7)
Retired	142 (47.7)
Household income (AUD) (***n*** = 298)	
≤ $40,000	90 (30.2)
69,000	76 (25.5)
99,000	60 (20.1)
≥ $100,000	72 (24.2)
Health care card (***n*** = 297)^⁑^	120 (40.4)
Socioeconomic position	
Quintile 1^‡^	36 (12.1)
Quintile 2	66 (22.0)
Quintile 3	52 (17.3)
Quintile 4	61 (20.3)
Quintile 5^†^	85 (28.3)
BMI, mean ± SD	27.9 ± 5.2
hs-CRP, mean ± SD (***n*** = 263)	2.2 ± 3.7
Vitamin D (nmol/L), mean ± SD (***n*** = 270)	65.6 ± 22.0
History of fracture (previous 5 years) (***n*** = 292)	45 (15.4)
Smoking status (***n*** = 298)	
Current smoker	13 (5.2)
Ex-smoker	113 (44.8)
Never	126 (50.0)
Comorbidities	
Cardiovascular disease	44 (14.8)
Diabetes	23 (7.7)
Cancer	18 (6.0)
Asthma	28 (9.4)
Arthritis	102 (34.2)
Osteoporosis	49 (16.3)
Sarcopenia (probable)	62 (20.7)
Chronic back pain	95 (31.9)
Depression/anxiety	42 (14.1)
Other	52 (17.4)
Number of comorbidities	
None	68 (22.7)
1	82 (27.5)
2	70 (23.6)
3	42 (14.1)
≥ 4	36 (12.1)

*AUD*, Australian dollars; *BMD*, bone mineral density; *hs-CRP*, high-sensitivity C-reactive protein; *SD*, standard deviation

^‡^Most disadvantaged socioeconomic quintile

^†^Least disadvantaged socioeconomic quintile

^⁑^In Australia, a health care card is a government-issued concession card that provides eligible individuals (usually those on low incomes) with reduced costs for prescription medicines, utilities, and certain health services

### Study outcomes

#### Bone mineral density and appendicular lean mass

The overall cohort had a mean BMD score of −1.38 ± 1.06 and an ALM/h^2^ score of 7.18 ± 1.33. There were no statistically significant associations between BMD values across any socioeconomic categories in the unadjusted model. When the model was adjusted for age, sex, and other variables, having post-secondary education was associated with higher BMD scores (β = 0.29; 95% CI: 0.01 to 0.58; *p* = 0.044) compared to having an education level of secondary school or less. Similarly, there were no statistically significant differences in ALM/h^2^ scores across any of the socioeconomic categories in the unadjusted analyses, except for area-level SES. When adjusted for using the same model, compared to having an education level of secondary school or less, those who had completed post-secondary education had higher ALM/h^2^ (β = 0.29; 95% CI: 0.08 to 0.51; *p* = 0.010), as did those in the highest income level group (β = 0.20; 95% CI: 0.01 to 0.39; *p* = 0.042). Table 2 presents mean (SD) scores and associations between socioeconomic position and BMD and ALM/h^2^.

**Table 2:** Associations between socioeconomic status and body composition measurements

Outcomes	SES variable	Mean ± SD	Unadjusted		Adjusted*	
			Coefficient (95% CI)	***P***-value	Coefficient (95% CI)	***P***-value
Bone mineral density (g/cm^2^)	Education					
	Secondary school or less	−1.48 ± 1.07	*(Reference)*	-	*(Reference)*	-
	Post-secondary school	−1.35 ± 1.06	0.14 (−0.14, 0.41)	0.336	**0.29 (0.01, 0.58)**	**0.044**
	Income level^#^					
	Low	−1.35 ± 1.11	*(Reference)*	-	*(Reference)*	-
	High	−1.43 ± 1.01	−0.08 (−0.33, 0.16)	0.500	−0.07 (−0.32, 0.17)	0.558
	Employment status					
	Unemployed/Retired	−1.36 ± 1.12	*(Reference)*	-	*(Reference)*	-
	Employed	−1.43 ± 0.93	−0.07 (−0.33, 0.19)	0.596	−0.23 (−0.53, 0.08)	0.139
	Health care card					
	Yes	−1.37 ± 1.11	*(Reference)*	-	*(Reference)*	-
	No	−1.39 ± 1.04	−0.02 (−0.27, 0.23)	0.860	0.02 (−0.25, 0.29)	0.880
	Area-level SES					
	Low (bottom 5 deciles)	−1.30 ± 1.07	*(Reference)*	-	*(Reference)*	-
	High (top 5 deciles)	−1.40 ± 1.06	−0.10 (−0.38, 0.18)	0.489	0.09 (−0.20, 0.37)	0.537
Appendicular lean mass/height^2^ (kg/m^2^)	Education					
	Secondary school or less	7.02 ± 1.31	*(Reference)*	-	*(Reference)*	-
	Post-secondary school	7.24 ± 1.33	0.21 (−0.13, 0.56)	0.225	**0.29 (0.08, 0.51)**	**0.010**
	Income level^#^					
	LowfAre you able to bold the regression scores and p values in the table that are significant	7.11 ± 1.38	*(Reference)*	-	*(Reference)*	-
	High	7.27 ± 1.27	0.16 (−0.15, 0.46)	0.315	**0.20 (0.01, 0.39)**	**0.042**
	Employment status					
	Unemployed/Retired	7.16 ± 1.30	*(Reference)*	-	*(Reference)*	-
	Employed	7.23 ± 1.39	0.08 (−0.25, 0.40)	0.644	0.14 (−0.10, 0.38)	0.252
	Health care card					
	Yes	7.16 ± 1.42	*(Reference)*	-	*(Reference)*	-
	No	7.19 ± 1.19	−0.04 (−0.35, 0.27)	0.813	0.19 (−0.02, 0.39)	0.073
	Area-level SES					
	Low (bottom 5 deciles)	7.57 ± 1.30	*(Reference)*	-	*(Reference)*	-
	High (top 5 deciles)	7.05 ± 1.32	**−0.51 (−0.86, −0.17)**	**0.004**	0.08 (−0.14, 0.30)	0.463

*CI*, confidence intervals; *SES*, socioeconomic status

*Adjusted for age, sex, born in Australia (yes/no), body mass index, multimorbidity (yes/no), smoking status (yes/no), high-sensitivity C-reactive protein (hs-CRP), and/or vitamin D level

^#^Low income was defined as < $70,000AUD and high income as > $70,000AUD (the average annual income for all employees in Victoria in 2018 was approximately $68,600AUD)

Regression coefficients represent the mean difference in the outcome variable between sociodemographic variables (e.g. high vs. low income). A positive coefficient indicates a higher mean score in the outcome associated with the respective SES characteristic, whereas a negative coefficient indicates a lower mean score. *Bold text* indicates statistical significance at *P*-value < 0.05

#### Muscle strength

The mean hand grip strength score of the cohort was 33.6 kg ± 9.65. Higher education level (β = 4.46; 95% CI: 2.01 to 6.91; *p* < 0.001), higher income (β = 3.44; 95% CI: 1.25 to 5.62; *p* = 0.002), and not having a health care card (β = 3.87; 95% CI: 1.67 to 6.08; *p* = 0.001) were significantly associated with higher handgrip strength scores in participants. These associations were slightly attenuated after adjusting for age, sex, and other characteristics, although they remained significant. The mean time to complete the five-times sit-to-stand test for lower limb strength was 13.12 s ± 10.99. Lower limb strength was also significantly greater in participants who had completed post-secondary education than those with less years of education in both unadjusted (β = −4.00; 95% CI: −6.86 to −1.13; *p* = 0.006) and adjusted (β = −2.54; 95% CI: −5.48 to −0.39; *p* = 0.039) analyses. Participants without a health care card had significantly greater lower limb strength compared with those with a health care card (β = −3.62; 95% CI: −6.19 to −1.04; *p* = 0.006); however, this association was no longer statistically significant in adjusted analyses. Table 3 presents associations between socioeconomic position, muscle strength, and physical function measurements.

**Table 3:** Associations between socioeconomic position, muscle strength, and physical function measurements

Outcomes	SES variable	Mean ± SD	Unadjusted		Adjusted*	
			Coefficient (95% CI)	***P***-value	Coefficient (95% CI)	***P***-value
Handgrip strength (kg)	Education					
	Secondary school or less	30.31 ± 8.83	*(Reference)*	-	*(Reference)*	-
	Post-secondary school	34.77 ± 9.68	**4.46 (2.01, 6.91)**	** < 0.001**	**2.55 (0.57, 4.52)**	**0.012**
	Income level^#^					
	Low	32.08 ± 9.22	*(Reference)*	-	*(Reference)*	-
	High	35.52 ± 9.87	**3.44 (1.25, 5.62)**	**0.002**	**2.07 (0.37, 3.78)**	**0.018**
	Employment status					
	Unemployed/Retired	32.89 ± 10.03	*(Reference)*	-	*(Reference)*	-
	Employed	35.17 ± 8.59	2.28 (−0.08, 4.65)	0.058	0.62 (−1.52, 2.76)	0.569
	Health care card					
	Yes	31.31 ± 8.90	*(Reference)*	-	*(Reference)*	-
	No	35.18 ± 9.87	**3.87 (1.67, 6.08)**	**0.001**	**2.78 (0.94, 4.63)**	**0.003**
	Area-level SES					
	Low (bottom 5 deciles)	33.86 ± 9.93	*(Reference)*	-	*(Reference)*	-
	High (top 5 deciles)	33.51 ± 9.57	−0.35 (−2.90, 2.20)	0.786	0.95 (−0.99, 2.89)	0.337
Five-times sit-to-stand test (seconds)	Education					
	Secondary school or less	16.10 ± 13.75	*(Reference)*	-	*(Reference)*	-
	Post-secondary school	12.10 ± 9.69	**−4.00 (−6.86, −1.13)**	**0.006**	**−2.54 (−5.48, −0.39)**	**0.039**
	Income level^#^					
	Low	14.19 ± 12.00	*(Reference)*	-	*(Reference)*	-
	High	11.83 ± 9.51	−2.35 (−4.91, 0.20)	0.071	−0.72 (−3.23, 1.80)	0.575
	Employment status					
	Unemployed/Retired	14.00 ± 11.64	*(Reference)*	-	*(Reference)*	-
	Employed	11.34 ± 9.35	−2.66 (−5.37, 0.06)	0.055	−0.01 (−3.13, 3.13)	0.998
	Health care card					
	Yes	15.32 ± 12.33	*(Reference)*	-	*(Reference)*	-
	No	11.71 ± 9.85	**−3.62 (−6.19, −1.04)**	**0.006**	−1.27 (−4.00, 1.46)	0.362
	Area-level SES					
	Low (bottom 5 deciles)	14.76 ± 13.18	*(Reference)*	-	*(Reference)*	-
	High (top 5 deciles)	12.57 ± 12.57	−2.19 (−5.11, 0.73)	0.140	−1.05 (−4.00, 1.90)	0.485
Gait speed (m/sec)	Education					
	Secondary school or less	1.10 ± 0.31	*(Reference)*	-	*(Reference)*	-
	Post-secondary school	1.27 ± 0.30	**0.17 (0.09, 0.25)**	** < 0.001**	**0.08 (0.01, 0.15)**	**0.030**
	Income level^#^					
	Low	1.17 ± 0.31	*(Reference)*	-	*(Reference)*	-
	High	1.30 ± 0.29	**0.13 (0.06, 0.20)**	**< 0.001**	**0.09 (0.04, 0.15)**	**0.002**
	Employment status					
	Unemployed/Retired	1.18 ± 0.32	*(Reference)*	-	*(Reference)*	-
	Employed	1.33 ± 0.24	**0.14 (0.07, 0.22)**	**< 0.001**	0.04 (−0.04, 0.11)	0.325
	Health care card					
	Yes	1.12 ± 0.30	*(Reference)*	-	*(Reference)*	-
	No	1.30 ± 0.29	**0.18 (0.11, 0.25)**	**< 0.001**	**0.10 (0.03, 0.16)**	**0.004**
	Area-level SES					
	Low (bottom 5 deciles)	1.17 ± 0.34	*(Reference)*	-	*(Reference)*	-
	High (top 5 deciles)	1.24 ± 0.29	0.07 (−0.01, 0.15)	0.089	0.05 (−0.02, 0.12)	0.165
Short Physical Performance Battery (0–12)	Education					
	Secondary school or less	10.52 ± 1.81	*(Reference)*	-	*(Reference)*	-
	Post-secondary school	11.27 ± 1.29	**0.75 (0.35, 1.15)**	**< 0.001**	0.34 (−0.02, 0.71)	0.067
	Income level^#^					
	Low	10.85 ± 1.63	*(Reference)*	-	*(Reference)*	-
	High	11.39 ± 1.18	**0.54 (0.20, 0.89)**	**0.002**	0.21 (−0.11, 0.52)	0.198
	Employment status					
	Unemployed/Retired	10.88 ± 1.62	*(Reference)*	-	*(Reference)*	-
	Employed	11.52 ± 0.95	**0.64 (0.28, 1.01)**	**0.001**	0.26 (−0.13, 0.65)	0.192
	Health care card					
	Yes	10.57 ± 1.65	*(Reference)*	-	*(Reference)*	-
	No	11.42 ± 1.23	**0.85 (0.50, 1.19)**	**< 0.001**	**0.42 (0.08, 0.75)**	**0.016**
	Area-level SES					
	Low (bottom 5 deciles)	10.62 ± 1.86	*(Reference)*	-	*(Reference)*	-
	High (top 5 deciles)	11.25 ± 1.26	**0.64 (0.25, 1.03)**	**0.001**	0.22 (−0.14, 0.58)	0.231
Leg power (watts)	Education					
	Secondary school or less	286.63 ± 112.82	*(Reference)*	-	*(Reference)*	-
	Post-secondary school	364.77 ± 145.54	**78.13 (38.83, 117.44)**	**< 0.001**	**41.65 (11.33, 71.97)**	**0.007**
	Income level^#^					
	Low	315.09 ± 134.02	*(Reference)*	-	*(Reference)*	-
	High	383.11 ± 143.17	**68.02 (33.64, 102.40)**	**< 0.001**	**45.01 (19.09, 70.94)**	**0.001**
	Employment status					
	Unemployed/Retired	317.49 ± 144.16	*(Reference)*	-	*(Reference)*	-
	Employed	404.01 ± 117.78	**86.52 (50.37, 122.67)**	** < 0.001**	**36.34 (3.12, 69.55)**	**0.032**
	Health care card					
	Yes	284.15 ± 118.44	*(Reference)*	-	*(Reference)*	-
	No	385.65 ± 142.17	**101.50 (67.73, 135.27)**	**< 0.001**	**69.97 (42.37, 97.38)**	**< 0.001**
	Area-level SES					
	Low (bottom 5 deciles)	334.31 ± 148.99	*(Reference)*	-	*(Reference)*	-
	High (top 5 deciles)	348.17 ± 139.44	13.86 (−26.27, 53.99)	0.497	19.87 (−10.43, 50.18)	0.198

*CI*, confidence intervals; *SES*, socioeconomic status

*Adjusted for age, sex, born in Australia (yes/no), body mass index, multimorbidity (yes/no), smoking status (yes/no), high-sensitivity C-reactive protein (hs-CRP), and/or vitamin D level

^#^Low income was defined as < $70,000AUD and high income as > $70,000AUD (as the average annual income for all employees in Victoria in 2018 was approximately $68,600AUD)

Regression coefficients represent the mean difference in the outcome variable between sociodemographic variables (e.g. high vs. low income). A positive coefficient indicates a higher mean score in the outcome associated with the respective SES characteristic, whereas a negative coefficient indicates a lower mean score. *Bold text* indicates statistical significance at *P*-value **< 0.05**

#### Physical function measures

The average gait speed in the cohort was 1.23 m/second ± 0.31. All SES variables were significantly associated with higher gait speed in unadjusted models, except IRSAD status. When adjusting for age, sex, and other variables, post-secondary education (β = 0.08; 95% CI: 0.01 to 0.15; *p* = 0.030), higher income (β = 0.09; 95% CI: 0.04 to 0.15; *p* = 0.002), and not having a health care card (β = 0.10; 95% CI: 0.03 to 0.16; *p* = 0.004) were significantly associated with higher gait speed in participants (Table 3). Similarly, all SES variables were significantly associated with higher SPPB scores in unadjusted models; however, these associations only remained statistically significant for health care card ownership in adjusted analyses (β = 0.42; 95% CI: 0.08 to 0.75; *p* = 0.016). Mean leg power in the whole cohort was 344.65 watts ± 141.76. Post-secondary education, high income, being employed, and not having a health care card were all significantly associated with greater leg power in both unadjusted and adjusted analyses (Table 3).

## Discussion

The influence of socioeconomic position on chronic disease is highly recognised in both research and clinical practice. This study investigated the associations between social disadvantage and musculoskeletal health, focusing on diagnostic measurements of osteoporosis and sarcopenia. We found clinically meaningful and statistically significant differences in BMD, muscle strength, and physical function measures according to various socioeconomic factors, suggesting that socially disadvantaged groups are at greater risk of developing osteoporosis and sarcopenia.

Education and income are the two most commonly used indicators of SES. Participants who had completed post-secondary education had significantly higher BMD and ALM/h^2^ values, stronger handgrip strength, faster gait speed, and greater leg power than those who had completed secondary education or lower. Our findings are consistent with previous cross-sectional and longitudinal studies that have shown that lower education levels are associated with lower BMD, higher odds of osteopenia/osteoporosis, and increased risk of fragility fracture in older men and women [19, 29–31]. Multiple cross-sectional analyses of large population-based studies across Australia and Europe have also reported that community-dwelling older adults with greater educational attainment are more likely to have stronger handgrip and more muscle mass and less likely to have probable sarcopenia than those with lower levels of education [22, 23, 32]. These associations may reflect the long-term benefits of higher SES, such as improved overall health outcomes.

Interestingly, while higher income was associated with enhanced muscle and physical function outcome measures, employment status was not. This finding appears contradictory, as employment and income are often presumed to correlate, and both are commonly used as indicators of socioeconomic position. One potential explanation for this paradoxical finding is the complexity of the relationship between employment and health in older adult populations. Employment has been shown to be associated with better physical health in middle-aged adults [33]; however, the mean age of this cohort was 66.8 years, and the majority were retired or not working (68.3%). Being unemployed at an older age does not indicate economic hardship or lower SES. Further to this, almost 20% of participants reported undertaking volunteer work, which may indicate being financially secure. Income is a more accurate measure of social disadvantage than employment status alone, as it more directly reflects an individual’s ability to afford private health insurance, hence greater access to health care services [34]. Similar distinctions have been observed in previous studies focusing on older adults where income, but not employment, was a robust predictor of generic health outcomes [35]. Another potential reason for this finding may relate to occupation type. Our study did not collect data on participants’ specific occupations, and prior research has highlighted that the type of employment can have important implications for musculoskeletal health. A study by *Brennan-Olsen *et al*.* showed that individuals employed in high-skilled white-collar occupations had significantly better muscle mass and strength outcomes than those in blue-collar or low-skilled white-collar roles [22].

In Australia, a health care card is a government-issued concession card that individuals who meet an income-based eligibility criteria (most commonly those receiving government support payments or those on low incomes) can apply for. It entitles holders to reduced costs for prescription medicines, as well as concessions on certain health services and essential utilities. Possession of a health care card is widely used as an indicator of socioeconomic disadvantage and is commonly used in Australian health research as a proxy measure for lower socioeconomic status and increased vulnerability. In this study, ownership of a health care card was associated with worse outcomes across several domains, including ALM/h^2^, grip strength, gait speed, and physical function. While this association has not previously been examined in the context of musculoskeletal health, health care card holders have been shown to experience poorer health and have a higher incidence of chronic health problems, contributing to increased health service utilisation [36, 37]. Our findings suggest that health care card status may serve as a particularly sensitive marker for musculoskeletal health, even after adjusting for age.

IRSAD was not significantly associated with any of the bone, muscle, or physical function outcomes in this study. This diverges from the consistent associations observed with individual-level SES indicators such as education, income, and health care card ownership. While population-based SES measures provide a broad assessment of community-level disadvantage, individual-level SES indicators offer more nuanced insights and may better reflect lived experiences and personal circumstances that encourage health care behaviours among individuals within the same geographic area.

Overall, 16.3% of participants (*n* = 49) had osteoporosis based on BMD values and 20.7% (*n* = 62) had probable sarcopenia, defined using the European Working Group on Sarcopenia in Older People (EWGSOP2) revised criteria for sarcopenia [38]. Both osteoporosis and sarcopenia are chronic conditions that often require the implementation of substantial lifestyle changes such as adhering to dietary or physical activity recommendations and, in some cases, following new medication regimens [39, 40]. These behavioural changes can be difficult to initiate and maintain over time and often require an adequate level of health literacy [41]. Health literacy is a multidimensional concept defined as ‘the personal characteristics needed for an individual to access, understand, appraise and use information about health and health care services to make decisions about health’ [42]. Few studies have investigated associations between health literacy and osteoporosis or sarcopenia [43]. Two randomised controlled trials evaluating health literacy-oriented behaviour change programs reported significant improvements in predictors of sarcopenia (timed up and go test; gait speed; grip strength), as well as increased physical activity levels and improved diet adherence for older adults compared to a standard care control group [44, 45]. However, there is a strong need for further research on associations between health literacy and musculoskeletal health [43].

### Strengths and limitations

We chose to focus on continuous measures of bone, muscle, and physical function rather than dichotomous classifications of the presence of osteoporosis or sarcopenia to capture a broader range of variation in musculoskeletal health. By analysing continuous outcomes, we were able to explore more subtle socioeconomic gradients that might be obscured by binary diagnostic thresholds and determine how socioeconomic disadvantage influences clinical precursors for sarcopenia and osteoporosis. We acknowledge that stratifying the prevalence of sarcopenia and osteoporosis by socioeconomic status could provide additional clinical context by directly highlighting disparities in diagnosed disease, which should be explored in future large-scale research. We utilised DXA to assess muscle and bone mass, which is considered the ‘gold standard’ for measuring BMD [7]. Muscle and physical function measures were also collected by trained physiotherapists/exercise physiologists and were conducted on multiple occasions to allow for familiarisation and ensure accuracy and reliability in assessments. Furthermore, we collected comprehensive demographic, lifestyle, and social data, which allowed us to control for these factors in our analysis. Despite these strengths, our study has several limitations that should be noted. The cross-sectional design prevented us from inferring causal inferences, and we cannot determine the directionality of the associations found in this study. A future longitudinal analysis will help us better understand how SES may influence changes in bone and muscle outcomes over time. Recruitment was conducted using a voluntary, convenience-based sampling approach, introducing potential selection bias, as participants were likely more health-conscious individuals with higher SES. However, recruitment was undertaken in one of the most socioeconomically disadvantaged areas in Victoria, as identified by the Australian Bureau of Statistics [24], which allowed for a broad representation of the socioeconomic factors examined in this study. The study also included many participants from diverse backgrounds who were not born in Australia (> 30%), which enhances the relevance of the findings to reflect the diversity of the Australian community. Lastly, the range of musculoskeletal outcomes in our sample was relatively narrow, likely due to the younger age of participants. This restricted variability may have reduced our ability to detect stronger associations, which could emerge in cohorts with a broader spread of bone, muscle, and function measures.

## Conclusion

This study has provided new evidence that socioeconomic disadvantage is associated with poorer bone, muscle, and physical function in older adults, and contributes to a deeper understanding of the mechanisms through which SES can influence musculoskeletal health. These findings highlight the importance of addressing social disadvantage in the development of preventive clinical strategies and updated policy approaches to reduce the risk of osteoporosis and sarcopenia.

## Data Availability

Access to this data should be discussed with the data custodian (JT).
